# Bacteremia among Jordanian children at Princess Rahmah Hospital: Pathogens and antimicrobial susceptibility patterns

**Published:** 2010-03

**Authors:** A Mohammad

**Affiliations:** Faculty of Nursing, Irbid National University, Irbid, Jordan

**Keywords:** Bacteremia, Child Fever, Antimicrobials, Drug resistance

## Abstract

**Objective:**

To investigate microorganisms causing bacteremia in Jordanian children and to assess their sensitivity to various groups of antimicrobials.

**Methods:**

A retrospective study was conducted on positive blood cultures taken from 378 children aged below 15 year, who sought medical attention at Princess Rahmah Hospital between January and December/2008.

**Results:**

Out of 4475 tested blood samples, 378 isolates were recovered from blood cultures. The male to female isolate ratio was (1.26:1.0). The most frequent pathogen found was *Staphylococcus aureus* (86.2%), followed by *Klebsiella spp.* (9%), *Escherichia coli* (1.9%), *Streptococcus spp.* (1.9%), *Pseudomonas spp.* (0.8%), and *Acinetobacter sp.* was found in only one culture (0.3%). The susceptibility rate of *S. aureus* was recorded the highest (99.6%) for vancomycin, and the lowest susceptibility rate (3.2%) was recorded for aztreonam.

**Conclusions:**

*Staphylococcus aureus* was the main isolate in bacteremic children, with all isolates demonstrating susceptibility to vancomycin. Overall, aztreonam resistance was near 97%, and this rate was not affected by sex and blood isolate type. This information should be considered when empirical therapy is recommended or prescribed for children with bacteremia.

## INTRODUCTION

Bacteremia is a common cause of morbidity and mortality in children (1) worldwide (2–5). Therefore, bacteremia continues to be a serious problem that needs immediate attention and treatment. For an accurate diagnosis and an appropriate choice of antimicrobials, blood culture, which usually takes a few days, is required. The empirical choice of antimicrobials for the treatment of bacteremia is guided by an awareness of previous culture reports. Up-to-date information on the local etiologic patterns and antimicrobial sensitivities is also important. Different factors contribute to the prognosis of the infection such as the type of microorganism, age, underlying disease and microorganism entry (6). However, bloodstream infections in hospitalized patients are usually attributable to the use of central venous lines.

In a prospective five-year study on 344 clinically significant episodes of pediatric septicemia the most common organisms found were *Salmonella spp.* (15.0%) followed by methicillin-resistant *S. aureus*. They reported that *Haemophilus influenzae* accounted for 2.0% of all episodes (7). Another study also reported that of 408 bacterial strains, *Salmonella spp.* were the most commonly isolated (23%), followed by *S. aureus* and *Acinetobacter-Moraxella* (8). The most frequent etiologic agents of bacteraemia cases include *Staphylococcus spp., Streptococcus spp., Enterobacter spp., Escherichia coli, Klebsiella pneumoniae and Pseudomonas spp*. (1, 6). However, etiologies of bacteremia and sensitivities have been changing over the past years (9, 10).

The rapid emergence of multidrug resistant bacteremia in developing countries is a new potential threat to the survival of newborn babies, who are in a poor health condition. Therefore, this study was conducted to assess the causative organisms and susceptibility pattern of bacteremia pathogens isolated from children in of 2008 (from Jan to Dec. 2008) at Princess Rahmah Hospital in Irbid, Jordan. The importance of this study is to aid clinicians to facilitate the empiric treatment and management of children with symptoms of bacteremia. Moreover, the data would also help authorities to formulate antibacterial prescription policies.

## MATERIALS AND METHODS

This retrospective study was conducted on 4475 blood specimens obtained from sick children (≤15 years of age) who attended the Princess Rahmah Hospital as outpatients or inpatients and were diagnosed with bacteremia between January and December, 2008. A total of 378 isolates were recovered from blood cultures, and the repeated positive blood cultures were not considered.

The microorganisms and antibacterial susceptibility data were obtained from the clinical microbiology laboratory records which filled in a prepared data sheet. Sampling process, culturing, bacterial identification and susceptibility testing for antimicrobials were as follows:

Blood specimens were collected in a blood culture bottle that contained 50 ml of tryptose phosphate broth and 0.02% polyanethol sulfonate (liquid). Following standard aseptic procedure, culture was incubated at 37°C for 24 hours prior to the isolation and identification of the bacteria.

Based on the Gram-staining characteristics of the bacteria growth in the blood culture bottle was subcultured onto MacConkey agar, Salmonella- Shigella agar, and Nutrient agar and/or blood agar plates. Bacteria isolated from colonies were further characterized by special biochemical and serological methods (11).

All isolates were tested for their susceptibilities to at least 8 out of 15 antimicrobials using antimicrobial diffusion discs (12). Bacterial sensitivity was tested for the following antimicrobials: Amikacin, Amoxicillin- Clavulanic acid, Ampicillin, Aztreonam, Cefaclor, Cefotaxime, Ceftazidime, Ceftriaxone, Cephlexin, Ciprofloxacin, Gentamicin, Imipenem, Pipracillin, Tobramycin and Vancomycin.

Data were analyzed statistically using SPSS (version 15 for Windows) program calculating the frequency and cross tabulations.

This protocol was approved by the Ethics Committee of the Ministry of Health in Jordan (MOH, REC, 08, 0057).

## RESULTS

In a 12-month duration (January to December 2008), a total of 378 out of 4475 blood samples of children below 15 years of age (55.8% male and 44.2% female) that gave a positive blood culture reaction were studied. Results showed that the majority of pathogens isolated were *Staphylococcus aureus* (86.2%), followed by *Klebsiella spp.* (9%), *Escherichia coli* (1.9%), *Streptococcus spp*. (1.9%), *Pseudomonas spp* (0.8%) and *Acinetobacter spp.* (0.3%) (Table 1). The antimicrobial susceptibility of bacteremia isolates for 15 selected antimicrobial agents used in this study are summarized in Table 2.

**Table 1:** Microbiological characteristics blood bacterial isolates.

	Bacteria	Sex		Total	(%)

		M	F		
1	*S. aurous*	176	150	326	86.2
2	*Klebsiella* spp.	22	12	34	9
3	*E. coli*	5	2	7	1.9
4	*Streptococcus* spp.	5	2	7	19
5	*P. aeruginosa*	2	1	3	0.8
6	*Acinetobacter* spp.	1	0	1	0.3
	Total	211	167	378	100.0

**Table 2:** Susceptibility rates of blood bacterial isolates to antimicrobial agents.

Drug	*S. aurous*		*Klebsiella* spp.		*E. coli*		*Streptococcus* spp.		*Pseudomonas* spp.		*Acinetobacter* spp.	

	n = 326		n = 34		n = 7		n = 7		n = 3		n = 1	

	No	S%	No	S%	No	S%	No	S%	No	S%	No	S%
Amoxicillin-Clavulanic acid	34	76.4	5	20	0	0	0	0	1	0	0	0
Amikacin	319	81.8	31	38.7	7	100.0	7	28.5	3	100.0	1	0
Ampicillin	106	32	13	0	2	0	2	0	1	0	1	0
Aztreonam	311	3.2	30	16.6	7	71.4	5	100.0	3	33.3	1	100.0
Ceftazidime	276	25	29	41.3	7	85.7	6	16.6	3	33.3	1	100.0
Cefaclor	7	85.7	2	0	1	100.0	0	0	0	0	0	0
Cephlexin	83	13.2	9	33.3	1	100.0	0	0	0	0	0	0
Ciprofloxacin	262	79.2	23	91.3	7	85.7	5	100.0	2	100.0	1	100.0
Ceftriaxone	289	44.9	31	16.1	7	71.4	6	83.3	3	0	1	0
Cefotaxime	262	69.4	32	12.5	6	66.6	7	85.7	3	66.6	0	0
Gentamicin	282	60.6	31	193	7	42.8	7	42.8	3	66.6	1	0
Imipenem	272	4.7	22	9.0	7	42.8	3	0	3	0	1	100.0
Pipracillin	303	52.1	30	13.3	7	57.1	5	100.0	3	66.6	1	0
Tobramycin	317	83.5	30	20.0	7	85.7	7	57.1	3	66.6	1	0
Vancomycin	295	99.6	24	20	7	14.2	6	83.3	0	0	0	0

n=Number of total isolates No=Number of tested isolates S%=Percentage of Sensitive isolates

The highest susceptibility rate of *S. aureus* was to vancomycin (99.6%), whereas the lowest susceptibility rate was to aztreonam (3.2%). The highest susceptibility rate of other isolates i.e. *Klebsiella spp*. were to ciprofloxacin 91.3%, *E. coli* and *Pseudomonas spp* demonstrated 100% susceptibility to amikacin. *Streptococcus spp*. and *Acinetobacter spp* demonstrated 100% susceptibility to both aztreonam and ciprofloxacin. Whereas, the lowest susceptibility rate for all other isolates, *Klebsiella spp. E. coli, Pseudomonas, Streptococcus spp.* and *Acinetobacter spp* was to ampicillin (0%). However, vancomycin was detected to give the highest susceptibility rate (91.5%) to a variety of bacteremia isolates, aztreonam exhibited the lowest susceptibility rate of 5.8% (Table 3).

**Table 3:** Susceptibility rate (%) of different blood bacterial isolates to antimicrobial agents.

Drug	Bacteria		

	Number of isolates	Susceptible	%
Vancomycin	332	304	91.5
Ciprofloxacin	300	243	81.0
Amikacin	370	287	77.5
Tobramycin	365	283	77.5
Cefaclor	10	7	70.0
Amoxicillin-Clavulanic acid	40	27	67.5
Cefotaxime	310	198	63.8
Gentamicin	331	185	55.8
Pipracillin	349	173	49.5
Ceftriaxone	431	160	37.1
Ampicillin	125	35	28.0
Ceftazidime	322	89	27.6
Cephlexin	93	15	16.1
Imipenem	358	27	7.5
Aztreonam	357	21	5.8

## DISCUSSION

This current study provides information regarding the main etiological agent *S. aureus* that causes bacteremia in children of both inpatients and outpatient and its antimicrobial susceptibility patterns. These results are in agreement with other studies that reported *S. aureus* as the most common bacteria isolated from blood of children (13–15).

An increase in the occurrence of *Staphylococcal* bacteremia is likely to be related to the increased use of intravascular catheters in medical care centers and puncture wounds (16, 17). The second most common organism causing bacteremia in this study was *klebsiella spp*. (9%). Similar results were reported by Rahman et al 2002 (14). However *Klebsiella* was the most common cause of neonatal sepsis in Karachi, Pakistan (18). However, in another studies conducted elsewhere, *Pseudomonas aeruginosa* was the most common organism (38.3%) followed by *Klebsiella* (30.4%) and *E coli* (15.6%) (19).

In this study, the occurrence of *E.coli, Streptococcus spp, Pseudomonas spp* and *Acinetobacter spp* were 1.9%, 1.9% 0.8% and 0.3% respectively. Higher occurrence of these blood isolates was reported in different literature (13, 19, 20).

The most effective antimicrobial agent against *Staphylococcus aureus* demonstrated in this study was vancomycin (99.6%), followed by cefaclor (85.7%), tobramycin (83.5%), ciprofloxacin (79.3%), amoxicillin-cavulanate (76.4%) and gentamicin (60.6%). Whereas low susceptibility rates were observed with ceftazidime (25%), cephlexin (13.2%), imipenem (4.7%) and aztreonam (3.2%). Similar results for vancomycin, ciprofloxacin and amoxicillin- cavulanate were reported in Jordan (21) and elsewhere (22). However, same authors reported high susceptibility rates of *S. aureus*, reaching 85–100% to both ceftazidime and gentamicin respectively.

In this study, *Staphylococcus aureus* showed resistant rate to ampicillin accounted for 67% which was lower than documented resistance rate of 85% conducted elsewhere (23).

However, the highest resistance rate (100%) to ampicillin observed in this study was for *Klebsiella, E.coli, Streptococcus spp, Pseudomonas spp* and *Acinetobacter spp*. Similarly low susceptibility rate (0%) of *Acinetobacter spp* to ampicillin has been reported in the literature (22).

in this study, the susceptibility rate of *Staphylococcus aureus* to cefaclor, amikacin, gentamicin and ceftriaxone was 85.7%, 81.8%, 60.6% and 44.9% respectively, which was lower than that reported by Shwe et al. 2002 (24).

The increasing rates of resistance in *Staphylococcus aureus* may be due to changes in the pathogen over the past years (10, 24). Ciprofloxacin showed to be the most effective drug for *Klebsiella spp*. with susceptibility rate of 91.3% which was higher than that reported previously in Jordan (25). However, the higher susceptibility rate of 100% to ciprofloxacin was reported in Tanzania (22).

At the same time, all blood isolates from selected children of this study showed highest susceptibility rate to vancomycin (91.5%) followed by ciprofloxacin (81.0%), amikacin (77.5%), tobramycin (77.5%), cefaclor (70.0%), amoxicillin-clavolanic acid (67.5%), cefotaxime (63.8%) and gentamicin (55.8%). Comparatively lower susceptibility rate to pipracillin (49.5%), ceftriaxone (37.1%), ampicillin (28%), ceftazidime (27.6%), cephlexin (16.1%), imipenem (7.5%) and aztreonam (5.8%) was demonestrated. These results are correspondent with the reported data (22, 26).

To conclude, the finding of this study showed that bacteremia in children is mainly caused by *Staphylococcus aureus* organisms, which develop resistance to commonly used antimicrobials. This emergence of multiple drug resistance calls for continuous monitoring and reviewing of antimicrobial policy in hospitals and the country at large. Therefore, this study is important for clinicians in order to facilitate the empiric treatment of children with symptoms of bacteremia. Moreover, the data would also help authorities to formulate antimicrobial prescription policies.
